# Association between heavy metal exposure and asthma in adults: Data from the Korean National Health and Nutrition Examination Survey 2008–2013

**DOI:** 10.1371/journal.pone.0319557

**Published:** 2025-03-10

**Authors:** Mijung Jang, Dohhee Kim, Seunghee Lee, KyooSang Kim

**Affiliations:** 1 Department of Research Institute, Seoul Medical Center, Seoul, Korea; 2 Department of Public Health, Graduate School, Korea University, Seoul, Korea; 3 Department of Occupational Environmental Medicine, Seoul Medical Center, Seoul, Korea; Endocrinology and Metabolism Population Sciences Institute, Tehran University of Medical Sciences, IRAN, ISLAMIC REPUBLIC OF

## Abstract

Risk factors for asthma include genetic, host, and environmental factors such as allergens, smoking, and exposure to chemicals. Heavy metals from air pollution or contaminated water and food can also trigger asthma. This study aimed to identify the biological exposure levels of blood lead, mercury, and cadmium, and determine the association of asthma with single and multiple exposures to these heavy metals using data from the Korean National Health and Nutrition Examination Survey (KNHANES) conducted between 2008 and 2013. A weighted analysis of 40,328 adults aged ≥ 20 years was conducted. Variables included blood heavy metal levels, health behaviors, demographic characteristics, and asthma status. Logistic regression was used to identify the association between the blood heavy metal levels and the odds ratio (OR) of asthma in adults. The overall asthma prevalence was 3.0%. The geometric mean values for blood lead, mercury, and cadmium were 2.14 μg/dL, 3.72 μg/L, and 0.96 μg/L, respectively. An association between asthma and high blood lead levels was observed, with the highest level group showing a statistically significant association. Blood mercury and cadmium were significantly associated with asthma in the highest quartile of blood levels. After adjusting for the demographic and health behavior variables, significant associations with asthma persisted for the highest quartiles of all heavy metals. Multiple exposures in the highest quartile also showed a significant association with asthma. This study demonstrated a significant association between blood heavy metal levels and asthma in adults, emphasizing the need to reduce exposure to lead, cadmium, and mercury as a preventive measure against asthma in adults.

## Introduction

Asthma is a common chronic disorder of the airways that occurs in all age groups and involves a complex interplay of underlying inflammation, including chronic airway inflammation, airway obstruction, and increased susceptibility to respiratory viral infections [1,2]. Approximately 300 million people worldwide, spanning all ages and ethnic groups, are affected by asthma, making it one of the most common chronic diseases, with an estimated prevalence of 16% globally [1]. The increased prevalence of asthma is associated not only with mortality but also with higher healthcare costs, including those related to medication, hospitalizations, and overall healthcare utilization [3,4]. Risk factors for asthma include genetic, host, and environmental factors such as allergens, smoking, and genetic predisposition [5]. Additionally, exposure to chemicals in various environments can affect the risk of developing asthma.

Among the chemicals, heavy metals can be found everywhere, including in soil, water, and air. Exposure to these metals occurs through air pollution or contaminated water and food. Heavy metals can trigger asthma due to exposure, acting as allergens [6]. Several studies have demonstrated that heavy metals can induce asthma [7,8], suggesting that they may affect immune function. Mercury exists in various forms and is a highly toxic heavy metal that can accumulate even at a very low concentration, posing significant health risks [9,10]. While some studies have reported an association between blood mercury levels and asthma in children [11], others have found no such relationship [12,13].

Cadmium, another heavy metal, is known for its carcinogenic properties and cumulative toxic effects, primarily targeting the kidneys and bones. However, no clear association between cadmium exposure and asthma has been established. Nonetheless, cigarettes contain cadmium, and smoking has been shown to be linked with asthma [14]. Lead is widely found in nature, with inorganic lead compounds being neurotoxic and potentially harmful to fetuses and children [9]. Several studies have examined the relationship between lead exposure and asthma, and blood lead levels have been found to be associated with bronchial hyperresponsiveness and allergens [15–17]. Various discussions remain regarding the association between these three heavy metals and asthma.

While numerous studies have established an association between heavy metal exposure and respiratory disease, most of this research has predominantly focused on pediatric populations. Children and adolescents have been extensively studied due to their heightened vulnerability to environmental pollutants and the long-term health implications of early exposure [18,19]. However, the impact of heavy metal exposure on adult respiratory health, particularly asthma, remains underexplored.

This difference is of particular concern, as adults may experience different exposure levels and exhibit different physiological responses to heavy metals compared to younger individuals. Furthermore, adults are likely to have accumulated exposure over a longer period, potentially exacerbating the health outcomes associated with such exposure. However, most existing research tends to examine the effects of individual metals, whereas real-world environmental exposure often involves simultaneous exposure to multiple metals. Such mixed exposures can interact in complex ways, potentially amplifying adverse health effects [20].

Emerging evidence suggests that simultaneous exposure to multiple heavy metals may synergistically contribute to the development and exacerbation of asthma in adults. Understanding these interactions is crucial for assessing the full impact of environmental pollutants on adult respiratory health. This study aimed to address these critical gaps by investigating not only the isolated impact of specific heavy metals on asthma in adults but also the combined effects of multiple metals, using data from the Korean National Health and Nutrition Examination Survey (KNHANES) from 2008 to 2013.

Using data from a representative population, this study aimed to determine the levels and differences in exposure to lead, mercury, and cadmium and identify the associations between asthma and single and multiple exposures to these heavy metals. This comprehensive approach is intended to enhance our understanding of the environmental determinants of asthma and guide targeted public health interventions.

## Materials and Methods

### Participants

This study was based on raw data from KNHANES conducted between January 2008 and December 2013. The KNHANES is a national survey annually conducted by the Korean Centers for Disease Control and Prevention (KCDC) to produce national statistics at the national, provincial, and metropolitan levels, assessing the health and nutritional status of the South Korean population. The survey comprises health, nutrition, and medical components.

The survey in these years was first stratified by resident registration population, province, and metropolitan levels. It was stratified into 26 strata for general areas and 24 for apartment areas, based on population ratios by sex and age group. Subsequently, 192 sample survey areas were selected, and all household members aged ≥ 1 year in 3,800 households were surveyed from January to December to obtain data.

The inclusion criteria for this study were adult participants, and adolescents were excluded from the analysis. Of the 53,829 cases collected during the survey, data from 40,328 adult participants were included in the weighted analysis. Missing or incomplete records were retained in the dataset, as appropriate statistical weighting was applied to ensure representativeness and minimize bias. All household members aged ≥ 1 year from 3,800 sampled households participated in the survey. Written informed consent was obtained from all participants

The study involved secondary data analysis using anonymized information. Therefore, it was exempt from requiring approval from the Seoul Medical Center’s Institutional Review Board (IRB No. 2024-07-003).

### Study variables

#### Heavy metal levels.

Blood samples for the measurement of heavy metals were collected using ethylenediaminetetraacetic acid (EDTA) tubes and analyzed (NeoDin Medical Research Institute, Seoul, South Korea). A minimum of 0.5 mL of whole blood was required for each analysis. For heavy metal analysis, whole blood samples were collected in Trace Element EDTA tubes (BD Co.) and handled under strict conditions to maintain sample integrity. Samples were transported in refrigerated conditions at 4°C to ensure stability.

Blood lead and cadmium levels were measured using graphite furnace atomic absorption spectroscopy (AAnalyst 600, PerkinElmer, Turku, Finland). Blood mercury levels were determined using a mercury analyzer and the gold amalgamation method (DMA-80, Milestone, Bergamo, Italy). For blood lead and cadmium, all measured data were used to estimate the distribution, excluding missing values. However, for blood mercury, the maximum value of 168.49 μg/L, was considered too large given the range of other measurements, which was then excluded from the analysis to estimate the distribution. For each blood heavy metal, the quartiles, geometric mean (GM), and geometric standard deviation (GSD) were calculated.

The limits of detection (LOD) reported during the study period were consistent across all measured heavy metals: 0.056 μg/L for cadmium, 0.12 μg/dL for lead, and 0.158 μg/L for mercury. To ensure analytical accuracy and consistency, internal quality assurance and control were conducted using commercially available standard reference materials (Lyphochek® Whole Blood Metals Control; Bio-Rad, Hercules, CA, USA). The inter-assay coefficients of variation (CV) ranged from 0.58% to 5.52% for blood lead, 0.97% to 7.78% for blood mercury, and 0.97% to 7.78% for blood cadmium, demonstrating reliable measurement precision throughout the study period. Although the Limit of Quantification (LOQ) was not explicitly reported, the consistent application of the LOD ensured reliable detection of analytes at low concentrations.

The NeoDin Medical Research Institute, accredited by the Korean Ministry of Labor, participated in international quality control programs, including the German External Quality Assessment Scheme and the Korea Occupational Safety and Health Agency (KOSHA). These measures ensured that the analyses adhered to international standards of accuracy and precision [21,22].

#### Multiple exposure levels.

Multiple exposure levels were created based on the three blood heavy metals as independent variables to create a model in which the effect of the blood heavy metals on asthma was evaluated. For the multiple exposure variables for blood heavy metals, heavy metals exposure scores were created by adding each of the standardized continuous heavy metal variables and then making them discrete variables with four values based on the quartiles of the scores.

#### Demographic variables.

The demographic variables included sex, age, region, occupation, education, body mass index (BMI), and household income. Age was classified into three groups: 20–39 years, 40–59 years, and 60 years or older. The region was categorized into Metropolitan Area and Provincial Region. The job was classified into white-collar jobs (professional, office, and service roles), blue-collar jobs (farmers, laborers, and manual laborers), and others (homemaker and student), and education was classified into middle school or below, high school, and college or above. Household income was divided into quartiles as categorized by the KNHANES.

#### Health behavior variables.

Smoking was categorized into never smoked, current smoker, and former smoker. Alcohol consumption was categorized into drinking and non-drinker. Walking exercise was categorized into exercising for more than 150 min and not exercising for less than 150 min per week.

#### Asthma.

The primary outcome variable in this study was the presence of asthma, defined based on self-reported physician diagnosis, as recorded in the KNHANES questionnaire. Participants were asked, “Have you ever been diagnosed with asthma by a physician?” Responses were coded as “yes” or “no.”

### Analysis methods

This study utilized data from the KNHANES, which employed a two-stage stratified cluster sampling method to select participants, ensuring a nationally representative sample. To account for the survey design, weights for each year of the 2008–2013 period were pooled according to the method suggested in the KNHANES guidelines. The pooled weights were used to generalize the findings to the entire South Korean population during the study period. Exposure to blood heavy metals was classified as “Lowest” if it was less than the first quartile (Q1), “Low” if it was between the first and second quartile (Q2), “High” if it was between the second and third quartile (Q3), and “Highest” if it was greater than the third quartile.

Geometric means and standard deviations of blood heavy metals were analyzed using T-tests or ANOVA. The association between blood heavy metals and asthma was analyzed using logistic regression, where the odds ratios (ORs) and 95% confidence intervals (CIs) were calculated. When using the heavy metal variables in the analysis, the lowest was set as the reference, and the OR was calculated. Three models (Models 1–3) were used to analyze the association between blood heavy metals and asthma. Model 1 considered only the corresponding heavy metal. Model 2 was adjusted for demographic variables in addition to Model 1. Model 3 additionally adjusted Model 2 for health behavior variables.

“System missing values,” “not applicable,” and “don’t know” for each variable were also treated as missing values according to the Complex Sample Analysis Guide in the KNHANES. After calculating the minimum sample size with G * power (version 3.1, Informer Technologies), we confirmed that our study met the required sample size of 134 participants. P values were two-tailed, with p < 0.05 considered statistically significant. Adjusted analyses were conducted to account for the complex sampling design of the KNHANES. The final weighted analyses were performed using SAS (version 9.4, SAS Institute), which is designed to handle complex survey data.

## Results

### General characteristics of the participants

The general characteristics of the participants after weighting are presented in Table 1. Demographic variables and health behavior variables were compared. The overall prevalence of asthma was 3.0%. The GM (GSD) values for blood lead, mercury, and cadmium were 2.14 μg/dL (1.006 μg/dL), 3.72 μg/L (1.009 μg/L), and 0.96 μg/L (1.008 μg/L), respectively (Table 2). Significant differences in blood heavy metal levels were observed across demographic and health behavior variables. Overall, males exhibited significantly higher levels of all three heavy metals compared to females. For blood lead, geometric means (GMs) were significantly higher among participants living in provincial regions compared to those in metropolitan cities. Additionally, higher blood lead levels were observed in males, older participants, those engaged in blue-collar jobs, individuals with lower education levels, lower income earners, smokers, drinkers, and those with a BMI ≥  25 kg/m². In contrast, blood mercury levels were notably higher among males, older participants, individuals with white-collar jobs, higher income earners, smokers, drinkers, and those with a BMI ≥  25 kg/m². Similarly, blood cadmium levels were significantly elevated among males, older participants, those living in provincial regions, individuals engaged in farming, fishing, or other manual labor jobs, lower income earners, smokers, drinkers, and those with a BMI ≥  25 kg/m².

**Table 1:** Characteristics of the study population (N =  40,328, weighted N = 38,232,401).

Variables		N (WN)	% (W%)
Sex	Male	17,412 (18,923,184)	43.2 (49.5)
	Female	22,916 (19,309,217)	56.8 (50.5)
Age	20–39	12,504 (14,977,611)	31.0 (39.2)
	40–59	15,028 (15,417,894)	37.3 (40.3)
	Over 60	12,796 (7,836,896)	31.7 (20.5)
Region	Metropolitan area	18,243 (18,268,266)	45.2 (47.8)
	Provincial region	22,085 (19,964,135)	54.8 (52.2)
Occupation	White collar job	11,976 (13,915,072)	32.4 (36.4)
	Blue collar job	9,804 (9,956,757)	26.5 (26.0)
	Others	15,198 (13,261,011)	41.1 (34.7)
Education	≤Middle school	14,056 (11,033,995)	34.9 (28.9)
	High school	12,343 (14,417,002)	30.6 (37.7)
	≥College	10,670 (11,811,994)	26.5 (30.9)
Household income	Low	9,828 (10,042,874)	24.4 (26.3)
	Low-middle	9,934 (9,515,068)	24.6 (24.9)
	High-middle	9,945 (9,343,909)	24.7 (24.4)
	High	9,937 (8,896,360)	24.6 (23.3)
Smoking	Former	10,596 (11,571,261)	26.3 (30.3)
	Never	4,632 (5,307,946)	11.5 (13.9)
	Current	21,874 (20,456,143)	54.2 (53.5)
Alcohol consumption	No	5,310 (4,112,354)	13.2 (10.8)
	Yes	31,779 (33,221,071)	78.8 (86.9)
BMI (kg/m^2^)	<25	25,792 (25,856,376)	64.0 (67.6)
	≥25	12,101 (12,239,486)	30.0 (32.0)
Walking exercise	No	27,724 (33,648,868)	68.8 (88.0)
	Yes	3,458 (3,589,906)	8.57 (9.4)
Asthma	No	5,473 (6,910,969)	13.6 (18.1)
	Yes	1,223 (1,039,908)	3.0 (2.7)

N, unweighted sample size; WN, weighted sample size; %, unweighted percent; W%, weighed percent.

**Table 2:** Geometric means and standard deviations of blood heavy metals according to the study population.

Variables		Lead (μg/dL)			Mercury (μg/L)			Cadmium (μg/L)		
		GM	GSD	p-value	GM	GSD	p-value	GM	GSD	p-value
Sex	Male	2.50	1.007	<0.001^†^	4.42	1.012	<0.001^†^	0.88	1.010	<0.001^†^
	Female	1.84	1.007		3.14	1.011		1.04	1.010	
Age	20–39	1.84	1.008	<0.001^†^	3.37	1.011	<0.001^†^	0.71	1.012	<0.001^†^
	40–59	2.36	1.007		4.23	1.012		1.13	1.009	
	Over 60	2.38	1.012		3.49	1.022		1.23	1.014	
Region	Metropolitan area	2.09	1.008	<0.001^†^	3.72	1.013	0.937	0.93	1.011	<0.001^†^
	Provincial region	2.20	1.008		3.72	1.014		0.98	1.011	
Occupation	White collar job	2.05	1.008	<0.001^†^	4.03	1.012	<0.001^†^	0.87	1.011	<0.001^†^
	Blue collar job	2.52	1.010		3.99	1.018		1.07	1.013	
	Others	1.98	1.009		3.24	1.014		0.99	1.013	
Education	≤Middle school	2.43	1.010	<0.001^†^	3.60	1.018	<0.001^†^	1.26	1.011	<0.001^†^
	High school	2.11	1.008		3.67	1.012		0.92	1.012	
	≥College	1.94	1.009		3.89	1.013		0.78	1.012	
Household income	Low	2.22	1.010	<0.001^†^	3.44	1.017	<0.001^†^	1.02	1.014	<0.001^†^
	Low-middle	2.17	1.011		3.62	1.015		0.98	1.014	
	High-middle	2.09	1.010		3.71	1.016		0.94	1.014	
	High	2.09	1.011		4.20	1.016		0.90	1.016	
Smoking	Former	2.33	1.013	<0.001^†^	4.08	1.021	<0.001^†^	0.82	1.016	<0.001^†^
	Never	1.89	1.007		3.27	1.011		0.93	1.01	
	Current	2.57	1.008		4.49	1.014		1.09	1.012	
Alcohol consumption	No	1.99	1.016	<0.001^†^	3.32	1.028	<0.001^†^	1.16	1.020	<0.001^†^
	Yes	2.16	1.006		3.77	1.009		0.94	1.008	
BMI (kg/m^2^)	<25	2.10	1.007	<0.001^†^	3.50	1.01	<0.001^†^	0.95	1.009	0.061
	≥25	2.24	1.009		4.22	1.014		0.98	1.014	
Walking exercise	No	2.12	1.006	<0.001^†^	3.71	1.010	0.144	0.96	1.008	0.098
	Yes	2.33	1.019		3.84	1.025		1.00	1.023	
Asthma	No	2.11	1.044	0.019^*^	3.74	1.053	0.669	0.98	1.055	0.003^*^
	Yes	2.41	1.041		3.89	1.077		1.23	1.059	

* p <  0.05 and ^†^p <  0.001 indicate statistical significance, determined by T-test or ANOVA.

**The p-value for differences in GM based on categorical variables was calculated using the F-statistic.

GM, Geometric mean; GSD, Geometric standard deviation.

### Association between asthma and the blood levels of heavy metals

The association between the presence of asthma and the blood levels of lead, mercury, and cadmium was analyzed (Table 3).

**Table 3:** Association between blood heavy metals and asthma.

Heavy metals	Range	Model 1		Model 2		Model 3	
		OR (95% CI)	p-value (trend^**^)	aOR (95% CI)	p-value (trend^**^)	aOR (95% CI)	p-value (trend^**^)
Lead quartile	Q1 (<1.62)	1	<0.001 (0.001) ^†^	1	<0.001 (0.001) ^†^	1	0.001 (0.003)^*^
	Q2 (>1.62–2.16)	1.49 (0.98–2.28)		1.59 (1.03–2.47)		1.54 (0.99–2.41)	
	Q3 (>2.16–2.84)	1.21 (0.77–1.90)		1.33 (0.82–2.14)		1.23 (0.76–1.97)	
	Q4 (>2.84)	2.27 (1.50–3.45)		2.65 (1.61–4.35)		2.55 (1.55–4.20)	
Mercury quartile	Q1 (<2.34)	1	0.037 (0.007)^*^	1	0.004 (<0.001)^†^	1	0.004 (<0.001)^†^
	Q2 (>2.34–3.61)	1.11 (0.71–1.73)		1.20 (0.76–1.89)		1.24 (0.79–1.95)	
	Q3 (>3.61–5.52)	1.31 (0.84–2.03)		1.57 (1.01–2.46)		1.58 (0.99–2.50)	
	Q4 (>5.52)	1.84 (1.18–2.87)		2.27 (1.41–3.66)		2.33 (1.44–3.77)	
Cadmium quartile	Q1 (<0.67)	1	<0.001 (<0.001)^†^	1	0.008 (0.001)^*^	1	0.017 (0.002)^*^
	Q2 (>0.67–1.44)	1.12 (0.72–1.76)		1.10 (0.70–1.73)		1.08 (0.69–1.72)	
	Q3 (>1.00–1.44)	1.52 (1.00–2.31)		1.42 (0.90–2.24)		1.43 (0.89–2.31)	
	Q4 (>1.44)	2.35 (1.58–3.49)		2.12 (1.34–3.35)		2.08 (1.28–3.39)	

* p <  0.05 and †p <  0.001 indicate statistical significance, determined by logistic regression analysis.

^a^Model 1 only considered heavy metals.

^b^Model 2 was adjusted for demographic variables.

^c^Model 3 was adjusted for demographic and health behavior variables.

Q1 is very low exposure to heavy metals.

OR, odds ratio; aOR, adjusted odds ratio.

For blood lead, participants in the highest quartile (Q4: > 2.84 μo/dL) had a significantly higher likelihood of having asthma compared to those in the lowest quartile (Q1: < 1.62 μ/dL). There were increasingly more associations in all quartiles from very low to higher blood lead concentrations in the study population, and the highest quartile was statistically significant (Model 1: OR =  2.27, 95%CI: 1.50–3.45; Model 2: adjusted OR [aOR] =  2.65, 95% CI: 1.61–4.35; Model 3: aOR =  2.55, 95%CI: 1.55–4.20). Even after adjusting for demographic factors and health behaviors, the association remained strong, suggesting that elevated blood lead levels may be independently associated with asthma prevalence.

A similar trend was observed for blood mercury. Participants in the highest quartile (Q4: > 5.52 μa/L) were significantly more likely to have asthma compared to those in the lowest quartile (Q1: < 2.34 μL/L). The aOR in Model 3 indicated a twofold increase in asthma risk in the highest quartile (aOR =  2.33, 95% CI: 1.44-3.77).

For blood cadmium, the highest quartile (Q4: > 1.44 μo/L) showed the strongest association with asthma prevalence. Participants in this quartile were more than twice as likely to have asthma compared to those in the lowest quartile (Q1: < 0.67 μL/L), with aORs of 2.12 (95% CI: 1.34–3.35) in Model 2 and 2.08 (95% CI: 1.28–3.39) in Model 3. Across all three metals, the highest quartile consistently demonstrated the most significant association with asthma prevalence.

### Association between exposure to multiple heavy metals and asthma

The association between multiple exposures and blood lead, mercury, and cadmium based on the asthma status was analyzed (Table 4).

**Table 4:** Association between exposure to multiple heavy metals and asthma.

Range	Model 1		Model 2		Model 3	
	OR (95% CI)	p-value (trend**)	aOR (95% CI)	p-value (trend**)	aOR (95% CI)	p-value (trend**)
Q1 (<0.34)	1	<0.001 (<0.001)^†^	1	<0.001 (<0.001)^†^	1	<0.001 (<0.001)^†^
Q2 (>0.34–0.98)	1.37 (0.90–2.10)		1.43 (0.92–2.22)		1.42 (0.91–2.23)	
Q3 (>0.98–3.36)	1.48 (0.94–2.35)		1.70 (1.03–2.80)		1.69 (1.01–2.84)	
Q4 (>3.36)	2.82 (1.85–4.32)		3.27 (2.00–5.38)		3.22 (1.93–5.36)	

*p <  0.05 and †p <  0.001 indicate statistical significance, determined by logistic regression analysis.

^a^Model 1 only considered heavy metals.

^b^Model 2 was adjusted for demographic variables.

^c^Model 3 was adjusted for demographic and health behavior variables.

Q1 is very low exposure to heavy metals.

OR, odds ratio; aOR, adjusted odds ratio.

In Model 1, which only considered the combined exposure levels of the three heavy metals, participants in the highest quartile (Q4: > 3.36) had significantly higher odds of asthma compared to those in the lowest quartile (Q1: < 0.34) (OR =  2.82, 95% CI: 1.85–4.32, p <  0.001). Meanwhile, the second quartile (Q2: 0.34–0.98) and the third quartile (Q3: 0.98–3.36) showed non-significant associations.

After adjusting for demographic variables, the association remained significant in the third and fourth quartiles. Participants in Q3 had an aOR of 1.70 (95% CI: 1.03–2.80), and those in Q4 had an aOR of 3.27 (95% CI: 2.00–5.38). In Model 3, which further adjusted for health behaviors such as smoking, alcohol consumption, and BMI, the associations remained robust in the highest quartiles. Participants in Q3 had an aOR of 1.69 (95% CI: 1.01–2.84), and those in Q4 had an aOR of 3.22 (95% CI: 1.93–5.36). Q2 did not show a significant association, but the risk increased in Q3 and Q4, indicating that cumulative exposure to multiple heavy metals exceeds a threshold level, leading to a heightened risk of asthma.

## Discussion

This study investigated the association between asthma and heavy metal exposure in adults. The KNHANES data were analyzed, where higher exposure to heavy metals was found to be significantly associated with asthma, even after adjusting for demographic, and health behavior variables. Besides the well-known association with lead, significant associations were established between asthma and exposure to cadmium and mercury. Associations observed for single heavy metals were also found to be associated with multiple exposures. The geometric means (GMs) of blood heavy metals in our study were consistent with previous findings from KNHANES and other national health surveys, despite differences in study population composition and statistical methods. In this study, the GMs were 2.14 μg/dL (1.006 μg/dL), 3.72 μg/L (1.009 μg/L), and 0.96 μg/L (1.008 μg/L) for blood lead, mercury, and cadmium, respectively. The highest levels of blood lead were noted among participants aged ≥ 60 years (GM: 2.38 μg/dL), those engaged in blue-collar jobs (GM: 2.52 μg/dL), and current smokers (GM: 2.57 μg/dL), indicating that occupational and lifestyle factors may play a significant role in lead exposure. Farmers and other manual laborers exhibited the highest cadmium levels (GM: 1.07 μg/L), likely reflecting occupational exposure. Current smokers also demonstrated significantly elevated cadmium levels (GM: 1.09 μg/L, p <  0.001), consistent with tobacco being a major source of cadmium. These findings underscore the role of demographic, occupational, and behavioral factors in influencing heavy metal exposure within the population.

Our findings align with previous studies. For example, a study analyzing biological exposures to lead, mercury, and cadmium based on KNHANES data reported similar GMs for these metals, despite differences in the study population and methodology [21]. Additionally, trends in heavy metal exposure in Korea have shown a decline over time, likely due to improved environmental regulations [22,23].

However, disparities by sex, age, and smoking status remain persistent. This is consistent with international studies, such as those based on NHANES data in the United States, which revealed that smoking is a significant contributor to cadmium exposure and that blood lead levels are consistently higher in males, older adults, and those with lower socioeconomic status [24,25].

The observed association between blood lead and asthma may be attributed to lead-induced oxidative stress and airway inflammation, which are known to exacerbate respiratory conditions. Similarly, mercury and cadmium, both recognized for their immunotoxic effects, may impair pulmonary function and contribute to asthma pathophysiology [26]. In adolescents, blood lead levels have been shown to significantly increase the risk of asthma. For every 1 μg/dL increase in blood lead level, asthma prevalence increased by 1.94 times, highlighting that even low-level lead exposure can significantly contribute to asthma development [27]. Similarly, a study on workers exposed to lead found a higher prevalence of asthma among those with elevated blood lead levels [28]. These findings support the hypothesis that long-term lead exposure induces systemic inflammation and damages the respiratory tract, potentially exacerbating asthma symptoms [29].

The association between blood cadmium and asthma was particularly evident at higher concentrations. While many relevant studies have focused on smokers, one study demonstrated that cadmium exposure from smoking alters the epithelial or mast cells in the respiratory tract, potentially worsening asthma severity [8,14]. This is consistent with the observation that the narrower airways of patients with asthma may increase pulmonary uptake of cadmium, thereby amplifying its effects. Furthermore, a study using NHANES data reported a significant association between blood cadmium levels and asthma, not only in smokers but also in non-smokers (OR =  2.84, 95% CI: 2.07–3.90) [14]. Similarly, a study of 5,912 Korean adults revealed that individuals with higher blood cadmium levels had a 1.55 times higher lifetime prevalence of diagnosed asthma compared to those in the lowest quartile (95% CI: 1.03–2.33) [8].

For mercury, significant associations with asthma were observed across all quartiles, particularly at higher concentrations. A study involving 1,800 Chinese adults found that a twofold increase in blood mercury levels was associated with a decline in pulmonary function, likely attributable to mercury intake through fish consumption [30]. These findings suggest that mercury may contribute to asthma development by impairing lung function and exacerbating airway inflammation, although further research is needed to clarify the underlying mechanisms.

Studies on multiple exposures to lead, cadmium, and mercury have revealed significant associations at higher concentrations, particularly in the third and highest quartiles, even after adjusting for demographic variables. This suggests that cumulative exposure to heavy metals independently contributes to asthma risk, particularly at elevated levels. In Model 3, which further adjusted for health behaviors, these associations remained robust in the highest quartiles. These findings indicate that the effect of multiple heavy metal exposures on asthma prevalence persists irrespective of both demographic and behavioral factors. Moreover, this highlights the importance of addressing cumulative environmental exposures in public health policies to mitigate asthma risk.

Other studies support the association between asthma and multiple environmental exposures, including particulate matter, ozone, and heavy metals [31–33]. For example, a study on long-term exposure to air pollution containing heavy metals found a significant association with asthma [28]. Another study involving Chinese adults demonstrated that high blood levels of several heavy metals negatively affected pulmonary function [32]. Similarly, animal experiments have shown that heavy metals can accumulate in the lungs and impair pulmonary function, further supporting the role of heavy metal exposures in respiratory diseases [33]. These studies provide strong evidence that both single and combined exposures to heavy metals significantly influence asthma risk in adults.

The mechanisms through which lead, cadmium, and mercury contribute to asthma risk are multifaceted. Lead primarily enters the body through inhalation of dust or vapor and is excreted by binding to red blood cells. However, it can affect multiple systems, including the central nervous, digestive, and cardiovascular systems, contributing to systemic inflammation that exacerbates respiratory conditions. Cadmium exposure in South Korea is predominantly through smoking, but it is also ingested via cereals, fish, and seaweed [34,35]. Increasing evidence links cadmium exposure to various adverse health outcomes, including cardiovascular disease, kidney damage, and cancer [36].

Conversely, mercury is primarily absorbed through the consumption of fish and shellfish, although other sources include dental amalgams and cosmetic products [30]. Mercury exposure is associated with mitochondrial dysfunction and increased oxidative stress, both of which can impair the body’s defense against air pollution [37]. High oxidative stress and impaired anti-inflammatory responses may contribute to chronic airway inflammation, a hallmark of asthma. These mechanisms underscore the need for targeted public health measures to reduce exposure to heavy metals and mitigate their impact on asthma prevalence in adults.

The primary findings of this study can be summarized as follows: The estimated levels of blood lead, mercury, and cadmium varied significantly across demographic variables such as sex, age, region, and household income, as well as health behaviors such as smoking and alcohol consumption. These variations were associated with asthma prevalence. By identifying the association between heavy metal exposure and asthma as an environmental risk factor, this study provides foundational information on the biological exposure levels of blood heavy metals and their implications for asthma prevention. A notable strength of this study is its utilization of data from a large, nationally representative sample of the South Korean population, enhancing the generalizability and reliability of the findings. Moreover, this study stands out by addressing the association between blood levels of heavy metals and asthma in adults, an area that has been relatively underexplored in prior research. Additionally, the inclusion of various demographic and behavioral risk factors in the analysis strengthens the validity of the observed associations.

However, this study is not without limitations. First, the cross-sectional nature of the design precludes the establishment of causal relationships or the determination of temporal sequences between heavy metal exposure and asthma onset. While self-reported asthma data were utilized, which may introduce recall bias, previous population-based studies have supported the validity of self-reported asthma. Future research should incorporate objective clinical measures, such as spirometry or physician-confirmed diagnoses, to enhance the accuracy of asthma classification. Second, the inability to adjust for specific confounders, such as dietary intake, may have influenced the results. Dietary variables are particularly important as food sources such as fish, seaweed, and cereals are significant contributors to mercury and cadmium exposure in South Korea. Including these variables in future studies would provide a more comprehensive understanding of the pathways through which heavy metals affect asthma risk. Additionally, heavy metal measurements in this study were limited to blood, which reflects short-term exposure rather than chronic exposure from long-term accumulation, such as in bones. Expanding the scope of exposure assessments to include alternative biomarkers such as hair or urine could offer valuable insights into long-term heavy metal exposure and its health impacts. Third, while occupation was included as a covariate in the analysis, its broad categorization limited the exploration of specific occupational exposures that may play a critical role in heavy metal exposure. Future studies should integrate more detailed occupational data to investigate the potential effects of job-related exposures on asthma development. Finally, these findings should be interpreted with caution due to the inherent limitations of the study design, including risks of bias and the challenge of generalizing results to populations outside South Korea. Future longitudinal studies are warranted to confirm these associations and establish causal pathways between heavy metal exposure and asthma.

Despite these limitations, this study provides valuable insights into the role of heavy metal exposure as a modifiable environmental risk factor for asthma in adults. The findings highlight the need for targeted public health interventions to reduce heavy metal exposure, particularly in vulnerable subgroups such as older adults, smokers, and those in high-exposure occupations. By addressing these gaps, future research can contribute to the development of more effective strategies for asthma prevention and control.

## Conclusion

This study identified a significant association between high-level exposure to heavy metals—lead, cadmium, and mercury—and an increased risk of asthma in a large cohort of Korean adults. These findings emphasize the urgent need for public health measures aimed at reducing exposure to these metals to mitigate asthma risk, particularly among vulnerable populations.

While no significant association was observed at lower exposure levels, the results suggest the presence of threshold effects, where higher concentrations of heavy metals are required to impact asthma onset. Future research should aim to define these threshold levels more precisely and use longitudinal data to establish causal relationships. Additionally, further investigations are needed to elucidate the biological mechanisms through which heavy metals contribute to asthma development, including their roles in oxidative stress, inflammation, and immune dysregulation.

By addressing these gaps, future studies can provide a stronger foundation for developing targeted interventions to reduce heavy metal exposure and its impact on respiratory health.
